# Adjuvant Hormonal Therapy in Postmenopausal Women with Breast Cancer: Physician's Choices

**DOI:** 10.1155/2012/849592

**Published:** 2012-12-09

**Authors:** Asim Jamal Shaikh, Shiyam Kumar, Sajjad Raza, Maria Mehboob, Osama Ishtiaq

**Affiliations:** ^1^Section of Oncology, Department of Medicine, The Aga Khan University Hospital, P.O. Box 30270, Nairobi 00100, Kenya; ^2^Department of Oncology, Sultan Qaboos University Hospital, Muscat, Oman; ^3^Dow Medical College, Karachi, Pakistan; ^4^The Aga Khan Medical College, Karachi, Pakistan; ^5^Department of Endocrinology, Shifa International Hospital, Islamabad, Pakistan

## Abstract

The choice of adjuvant hormonal therapy in postmenopausal women with hormone receptor positive breast cancer has remained a matter of controversy and debate. The variety of agents is available, with each claiming to be superior. This clinical survey was undertaken to get an impression of the physician's first choice of therapy in an attempt to find out what questions still need to be answered in the making of “standard of care.” A web-based clinical survey was sent to the cancer physicians around the world, and 182 physicians responded to the survey. Most were medical oncologists in a tertiary care hospital. 36.3% preferred Anastrozole, 35.2% Tamoxifen, and 22.2% Letrozole as their first choice. Data support (67.8%) and safety concerns (30%) were given as the main reasons for the choice, 63.7% switched their therapy, and 24% had to switch because of side effects. 73.6% used 5 years of adjuvant hormonal therapy, 6.6% for 7 years, and 4.4% for 10 years. 61.5% follow their patients 3 times monthly, and 73.2% used laboratory and radiological assessment at each followup. *Conclusion*. Physicians show disagreement over the choice and duration of hormonal therapy in this patient population. Clinical trials leading to firm recommendations to set standards from which patients benefit the most are needed.

## 1. Introduction

Breast cancer remains the leading cause of cancer related morbidity and mortality in women, worldwide. Every tenth new cancer diagnosed is that of breast, and nearly a quarter of cancers in the women are breast cancer. About 1.4 million new breast cancer cases are diagnosed every year, and the burden is going to rise tremendously [1].

The incidence of breast cancer rises with increasing age, about 80% of breast cancer occur in women above 50 years of age and nearly half in the age group range of 50 to 69 [2]. The majority of this postmenopausal women with early breast cancer are hormone, that is, Estrogen (ER) and progesterone (PR) receptor positive, thus hormonal therapy is the mainstay of the treatment for them. Hormone therapy, which essentially deprives the tumor cells of hormone, relies on the fact that the tumor cells from the breast retain the property of thriving in the presence of female sex hormones, and dispossession of which results in inhibition of cancer cell regrowth.

Hormonal therapy is confirmed to render at least a benefit of 47% risk reduction for recurrence and that of 26% for mortality. This benefit alone surpasses the combined gain obtained by all other interventions in the adjuvant setting [3]. Most noticeably, the hormonal therapy intervention benefits all the patients who are hormone positive, regardless of their tumor size and nodal status. 

Tamoxifen has traditionally been considered the gold standard for adjuvant hormonal therapy in hormone receptor positive breast cancer. The newer third generation aromatase inhibitors have challenged this unparalleled status of tamoxifen in postmenopausal women, in whom cessation of ovarian function leaves only peripheral conversion of steroids as the source of production of estrogen and therefore making it an attractive target to block. Data from randomized phase III trials has now shown for most available AIs to have better toxicity profile and at least equal, if not superior, efficacy than the traditionally used tamoxifen. The physicians who still prefer to use Tamoxifen quote unique mechanism of action with possible intrinsic benefits and low cost as the reason behind the choice [4]. While the advocates of the newer third generations AI cite greater safety and improved PFS including a possible overall survival benefit as strong reasons for shifting the gear in favor of AIs as initial therapy. There is now in addition a data to support the switch strategy, in which one agent is alternated with the other, to have actually more beneficial effects than traditionally used continuous single agent for five years [5].

This mix of emerging and existing data has understandably expanded the options for physician prescription; besides, the many unanswered or half answered questions regarding whether to use AIs, which AI, longer use and switching between AIs and Tamoxifen, has left the treating physician to use the best personal judgment in making a choice before solid clinical data are available [6]. With this background we conducted a survey to on practicing oncology physicians worldwide as to see the trends of prescriptions of hormonal treatment for postmenopausal women in early adjuvant setting. With the propose of identifying what questions still need to be answered unequivocally from clinical trials that would set the standards from which this, the majority, of breast cancer patients would benefit the most. 

## 2. Materials and Methods

A web page was developed, which asked physicians five main questions, after collecting data on their profile and country of practice. The questions asked about the choice of physicians hormonal agent in early adjuvant setting for the postmenopausal women without having a significant co-morbid, such as a history of osteoporosis, stroke, ischemic heart disease, and hyperlipidemia. The questionnaire also asked duration of use of hormonal therapy, “switching therapy,” laboratory investigations done on followup, and the frequency of follow up. The main questions were followed by subsections on why a particular choice was made. The webpage address was sent to contactable physicians whose emails were available in the oncology conference database. Institutional ethics committee permission was obtained. SPSS software was used to analyze and prepare the descriptive data.

## 3. Results

The survey was sent out to 522 physicians, a total of 182 physicians responded to the questionnaire, and the response percentage is 34.8%. Most of participants that are 142 (78.0%) were medical oncologists, 24 (13.2%) clinical oncologists, while 14 (7.8%) were radiation oncologists by profession. Seventy-five percent of those were males, and 25% were females. Approximately one-third (38.5%) of them were above 45 years of age while remaining participants were younger than 45; likewise one-third (33%) of them were in clinical practice for less than 5 years while remaining two-thirds have clinical experience of more than 5 years. Most of the responding physicians were working either as private physicians or in teaching hospitals (40.7% each). Participants were from most parts of the world, including Pakistan, India, Saudi Arabia, Middle east, UK, Europe, and USA. A detailed list of the participants is given in Table 1.

**Figure 1:**
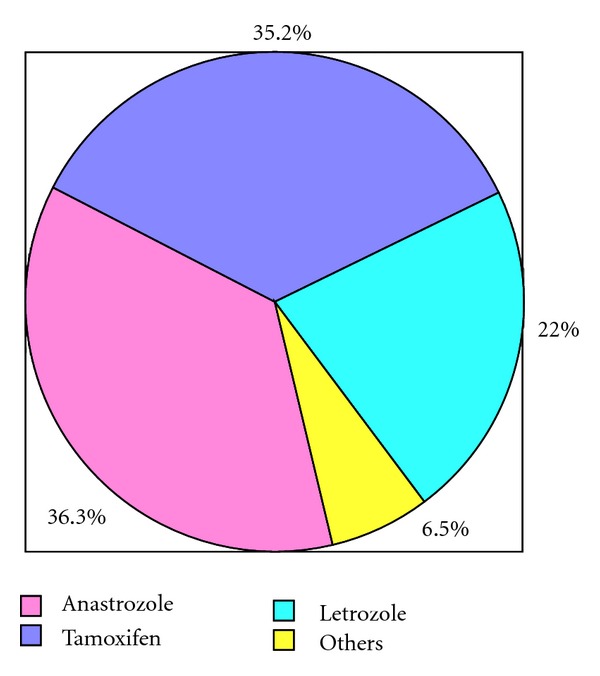
The choice of hormonal agent.

**Figure 2:**
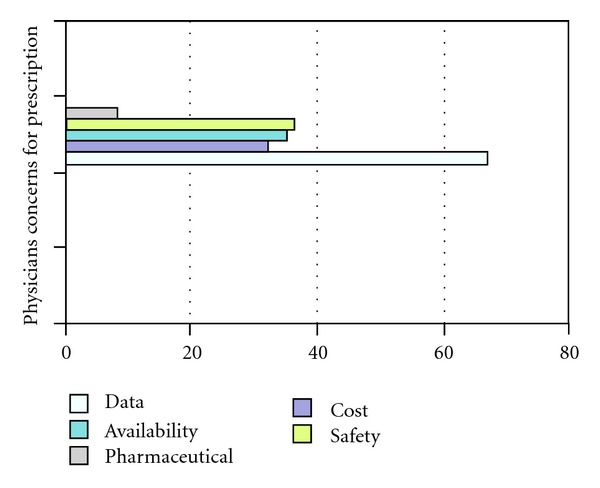
Consideration for prescription.

In response to question “*What is your first choice of hormonal agent in early adjuvant setting for receptor positive postmenopausal breast cancer patients, without significant comorbid like osteoporosis, DVT, heart disease*?” 36.3% chose Anastrozole as their choice of therapy, 35.2% favored tamoxifen, and 22% preferred Letrozole as their first choice. The choice of therapy by physicians is depicted in Figure 1. More than two-thirds (67%) of the participants gave the reason of having a robust clinical data behind their choice of therapy, while one-third stated concerns regarding safety as their primary motivation behind the choice of the agent of their preference. 31% physicians stated they consider cost, and 34% consider ease of availability as an additional factor in making a choice for the hormonal agents. About 8% stated that a trustworthy pharmaceutical company was the major factor behind the decision regarding the drug of their choice.  Figure 2 summarizes the reason considered for prescription by different physicians. In answer to the question “*Do you switch your patient from one agent to other during treatment, in a patient who does not have any progression of disease*?” 63.7% responding physicians said yes while 33% replied in negative. Amongst the physicians who switched to another agent 67.2% based their decisions on presence of data, and 24% had to switch the therapy due to problems encountered during the patient management. Of 33% of physicians who do not switch their patients from one agent to other 80% of them based their decisions on lack of data favoring the benefit of switching the therapy according to them. Medical oncologists switched therapy more often than a clinical or radiation oncologists (0.002). When the participants were asked about *the number of investigation do they undertake before starting the treatment of the patients in early adjuvant breast cancer treatment for postmenopausal women, without a major known comorbidity*, 73.6% replied that they do some investigations.  Table 2 summarizes the laboratory and radiological investigations frequently requested by the physicians on followup. Nearly two-thirds of physicians (61.5%) followed their patients 3–6 monthly during the 1st year of followup while 21% follow them every other month, 10% every month, and 2% follow their patients every 6 monthly. Most of physicians (73.6%) keep their patients for 5 years on chosen hormonal therapy; 8.8% want their patients to take hormonal therapy for 2 years, 6.6% for 7 years, and 4.4% for 10 years. Insignificant differences were also noticed based on designation, type, country, age, and years of practice.

## 4. Discussion

The concept of breast cancer's hormone sensitivity is more than a century old, when oophorectomies were shown to result in regression of advanced breast cancers [7]. Deriving benefits from the same concept, subsequent pharmacologic developments, and refining breast cancer subtypes, today hormonal therapies are the mainstay of hormone receptor positive breast cancers, which make up about 75% to 80% of the breast cancer population [8]. The data looking at effects of chemotherapy and hormonal therapy for early breast cancer on recurrence and 15-year survival by evaluation of all randomized trials from early breast cancer trialists group (EBCTG) suggest a 40% reduction in the risk of recurrence and 30% reduction in the risk of death with intervention from hormonal therapy, in women with hormone positive breast cancer, making hormone therapy, the single most powerful intervention, in terms of patient benefit [9]. 

Tamoxifen an oral nonsteroidal, antiestrogenic compound with an intrinsic proestrogenic activity was first discovered in 1966 and first approved for clinical use in 1977 for metastatic breast cancer. Tamoxifen has been one of the most commonly prescribed antineoplastic agents of all times [10]. Tamoxifen was found, through series of studies, to render sufficient therapeutic benefits without significant toxicity to justify the title of being gold standard for antiestrogen treatment in breast cancer [11]. The status of Tamoxifen as a gold standard has been significantly challenged by the newer generation of Aromatize inhibitors which in themselves are a robust group of antiestrogenic therapy [12]. The mechanism of action of AIs relies on the fact that most estrogen in the postmenopausal women comes from conversion of steroid hormones to estrogen. The conversion takes place primarily in the adrenals and at multiple other sites and is mediated by the aromatase enzyme. This enzyme is effectively blocked by the third-generation aromatase inhibitors.

The third-generation aromatase inhibitors first made their impact when they were compared to megestrol acetate in metastatic breast cancer (MBC) setting and showed superior time to progression (TTP) [13]. To date in at least five randomized Phase III studies done subsequently AIs demonstrated mostly superior response rate and time to progression compared to Tamoxifen, when used in metastatic breast cancer [14, 15].

 AIs have been clinically tested in adjuvant setting where they have unequivocally proved, in at least four phase III trials; that early adjuvant therapy with third-generation aromatase inhibitor in postmenopausal women with hormone positive breast cancer provides better disease-free survival (DFS) compared to the traditionally used Tamoxifen. All three available AIs Anastrozole, Letrozole, and Exemestane have been separately compared with Tamoxifen [16]. The intergroup Exemestane study studied switching to Exemestane after initial 2-3 years of Tamoxifen therapy in more than 4500 patients and showed improved disease free and a modest improvement in overall survival in ER positive women [17]. The followup study results of long-term use Letrozole versus placebo after 5 years of tamoxifen in the NCIC CTG MA.17 trial analysis were recently published and suggest that extended adjuvant Letrozole was superior to placebo in DFS and OS [18]. There are, however, serious considerations associated with the use of AIs in the form of increased expense and adverse impact on quality of life with increased bone loss and joint pains [19]. There have, thus, been calls for careful interpretation of complex scientific data available and to identify important subsets of population who are to actually derive the greatest benefit from the use of AIs [19, 20].

The existing and evolving knowledge and research on refinement in hormonal therapy for postmenopausal women generally is favoring the use of AIs, a longer duration of therapy and possibly for using the agents alternatively. However, the existing data appears not to have reached a threshold of generating distinct guidelines in favor of their use in this particular patient population, except for suggestions for the same, neither has been the duration of therapy, and the “switch strategy” reached a point where recommendation can be made unequivocally [21]. Controversies in setting up the end points, complexity of the data and crossover of the patients from one arm to the other are some of the factors responsible for the ambiguity [19].

Our survey clearly demonstrates the discord that is seen in general amongst the treating physicians. Although our survey has limitations in the form of number of participants and geographical variance to claim global representation; we, nevertheless, find that Tamoxifen is still prescribed as frequently in postmenopausal women with early stage breast cancer in adjuvant setting as are AIs. It seems that physicians favor Anastrozole over Letrozole, without obvious reason. The steroidal AI Exemestane is prescribed in the least of AIs. Switch therapy has been found to be sufficiently practiced amongst our survey population where close to 64% physicians actually do mention to switch the patients to Exemestane based on the data. Most physicians did not continue the therapy for more than 5 years. There were individual differences in the frequency of followups and laboratory or radiological investigations requested by physicians. It seems that in general there is a lack of clear guidelines available to physicians on how to follow the patients after they are over with the active adjuvant treatments that involve chemotherapy and radiation therapy. 

## 5. Conclusions

Physicians vary over choices on the preferred agents of hormonal therapy in postmenopausal women in whom there are no significant comorbid. They also differ quite significantly among the duration, switching the therapy, frequency of followup, and necessary laboratory and radiological investigations in such setting. In order to benefit the patients the most from what is believed to be corner stone of the therapy, further optimization of hormonal therapy with answers from clinical trials designed to identify the subgroups, benefitting the most from a specific hormonal therapy, shall perhaps set the standards.

## Figures and Tables

**Table 1:** Country of practice of physician.

Country of practice	no.	%
Pakistan	24	13.2
India	24	13.2
Turkey	18	9.9
Egypt	16	8.8
Saudi Arabia	8	4.4
Iran	8	4.4
UAE	6	3.3
UK	6	3.3
USA	6	3.3
Kuwait	6	3.3
France	6	3.3
Lebanon	4	2.2
Morocco	4	2.2
Italy	4	2.2
South Africa	2	1.1
Switzerland	2	1.1
Thailand	2	1.1
Algeria	2	1.1
Argentina	2	1.1
Brunei	2	1.1
Germany	2	1.1
Iraq	2	1.1
no. information	26	14.3

**Table 2:** Laboratory tests requested on followup.

Lab test	Yes number (%)	No number (%)
CBC	134 (73.6)	48 (26.4)
LFT	88 (48.4)	94 (51.6)
Ca^++^ + Vitamin D level	58 (31.9)	124 (68.1)
Thyroid functions	16 (8.8)	166 (91.2)
Echocardiogram	40 (22.0)	142 (78.0)
Doppler of lower legs	16 (8.8)	166 (91.2)
CA 15-3	54 (29.7)	128 (70.3)
BMD	124 (68.1)	58 (31.9)
Ultrasound pelvis	50 (27.5)	132 (72.5)
